# Quantitative Stain-Free and Continuous Multimodal Monitoring of Wound Healing *In Vitro* with Digital Holographic Microscopy

**DOI:** 10.1371/journal.pone.0107317

**Published:** 2014-09-24

**Authors:** Dominik Bettenworth, Philipp Lenz, Philipp Krausewitz, Markus Brückner, Steffi Ketelhut, Dirk Domagk, Björn Kemper

**Affiliations:** 1 Department of Medicine B, University Hospital Münster, Münster, Germany; 2 Center for Biomedical Optics and Photonics, University of Münster, Münster, Germany; 3 Biomedical Technology Center, University of Münster, Münster, Germany; Dalhousie University, Canada

## Abstract

Impaired epithelial wound healing has significant pathophysiological implications in several conditions including gastrointestinal ulcers, anastomotic leakage and venous or diabetic skin ulcers. Promising drug candidates for accelerating wound closure are commonly evaluated in *in vitro* wound assays. However, staining procedures and discontinuous monitoring are major drawbacks hampering accurate assessment of wound assays. We therefore investigated digital holographic microscopy (DHM) to appropriately monitor wound healing *in vitro* and secondly, to provide multimodal quantitative information on morphological and functional cell alterations as well as on motility changes upon cytokine stimulation. Wound closure as reflected by proliferation and migration of Caco-2 cells in wound healing assays was studied and assessed in time-lapse series for 40 h in the presence of stimulating epidermal growth factor (EGF) and inhibiting mitomycin c. Therefore, digital holograms were recorded continuously every thirty minutes. Morphological changes including cell thickness, dry mass and tissue density were analyzed by data from quantitative digital holographic phase microscopy. Stimulation of Caco-2 cells with EGF or mitomycin c resulted in significant morphological changes during wound healing compared to control cells. In conclusion, DHM allows accurate, stain-free and continuous multimodal quantitative monitoring of wound healing *in vitro* and could be a promising new technique for assessment of wound healing.

## Introduction

Epithelial wound healing is a common physiological process. In particular, within the gastrointestinal tract, there is persistent regeneration of epithelial cells to compensate physiological exfoliation of surface cells [1]. *Vice versa*, impaired wound healing has a tremendous pathophysiological implications in several conditions such as gastrointestinal ulcers [2], anastomotic leakage [3] venous or diabetic skin ulcers [4] and corneal ulcers [5]. Despite great advances in the pathophysiological concepts of wound healing, the molecular background is still incompletely understood and development of pharmacological agents to accelerate wound closure is required. However, evaluation of drug candidates is hampered since *in vivo* models can be complex and of limited availability [6]. Therefore, potential drug candidates are usually assessed in *in vitro* wound assays, such as the classical scratch assay established by Burk *et al.* [7]. Recently, more sophisticated cell culture systems have been introduced, more precisely elucidating the extent of migration and proliferation *in vitro* [8]. One example includes a silicone cell culture-inserts onto the cell culture surface generating two reservoirs (see also section ‘*Cell layer wound assays*’) that are separated by a 500 µm wall, which on removal leaves a well-defined border [9]. However, valid determination of cell migration commonly requires cell staining, e.g. Giemsa staining [10], [11] or transfection of the sample with fluorescent chromophores for cell tracking [12] which both require interaction with the sample.

Recently, bright field images and Zernike phase contrast images recorded with time-lapse video microscopy were established for analysis of wound healing assays *in vitro* [13]. Both techniques minimize the interaction between the imaging modality and the sample, and allow the quantification of the area change during cell migration into the wound either manually or computer assisted by image processing algorithms [14]. An electrically analysis approach based on automated impedance measurement during wound healing *in vitro* has been reported by Keese *et al*. [15]. This non-imaging approach allows the quantitative temporal observation of large areas covered with cells. However, these label-free modalities lack the ability for simultaneous assessment of cellular morphology and mass alterations.

Digital holographic microscopy (DHM), a variant of quantitative phase microscopy, enables not only stain-free quantitative phase contrast imaging but also assessment of cell thickness and tissue density by measuring optical path length delay [16]–[18]. Recently, it has been demonstrated that DHM provides quantitative monitoring of physiological processes through structural analysis and functional imaging which, for example, gives new insight into signaling of cellular water permeability [19], [20], cell morphology changes due to toxins [21]–[24] and infections [25]–[27] as well as micro-calcification, cancer and inflammation mediated tissue alterations [12], [28] and bacteria and mammalian single cell growth [29]–[32].

The aim of this study was to evaluate DHM as a novel method to accurately assess wound healing *in vitro* in a stain-free and continuous manner and to test its properties for quantitative determination of cellular changes upon cytokine stimulation. To the best of our knowledge, this is the first time a multi-parameter analysis of cellular growth and motility from quantitative phase images during epithelial wound healing has been performed.

## Results

### Visualization and assessment of epithelial wound healing by white-light microscopy and DHM

**Figure 1:**
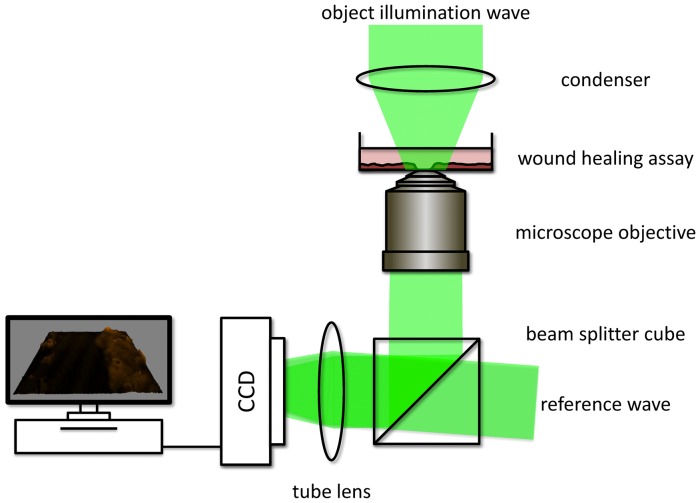
Utilized off-axis setup for digital holographic microscopy (DHM). A laser beam is divided by a beam splitter into an object wave, illuminating the specimen through a condenser and an undisturbed reference wave. The object wave interferes with the slightly tilted reference wave on a charge coupled device sensor (off-axis geometry). Morphological changes of the biological specimen lead to changes of the optical path length of the object wave, which are coded in the resulting interference pattern (digital hologram).

**Figure 2:**
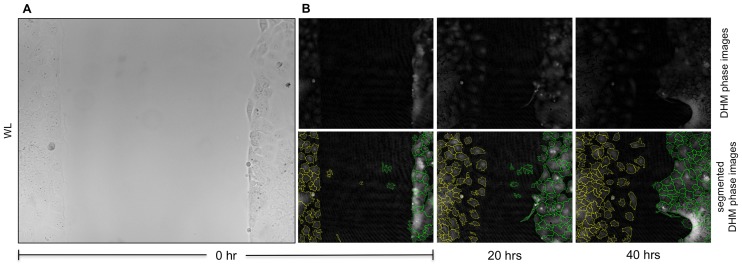
Visualization of epithelial wound healing by white light microscopy and DHM. (**A**) Conventional white light microscopy is hardly able to visualize outer borders of Caco-2 cells. (**B**) Phase contrast images provided by DHM (upper row) enable recognition of cell outlines, which are depicted by segmented DHM phase contrast images (lower row).

To evaluate the potential of quantitative phase imaging with DHM for monitoring of epithelial wound healing *in vitro*, Caco-2 cell wound assays were analyzed (see Figure 1 and description in the section ‘*Cell layer wound assays*’) and results compared to microscope images acquired by white light illumination. Mimicking different physiological situations, cells were stimulated with either epidermal growth factor (EGF, cell population of the assays in Figure 2), which is known to stimulate epithelial cell migration [33], [34] or treated with mitomycin c which is a well-known cell cycle inhibitor (left cell population of the assays in Figure 2) [34].

Figure 2A indicates that conventional visualization of Caco-2 cells by white-light microscopy is of limited feasibility due to the low contrast. In contrast, segmentation of quantitative DHM phase contrast images by image processing with the software cell profiler allowed delineation of the cell-covered surface and partly enabled single cell identification at different time points (Figure 2B). Assessment of wound closure 40 h after starting of the experiment revealed markedly accelerated migration of EGF-stimulated Caco-2 cells into the cell free gap in comparison to mitomycin c treated cells (Figure 2B, Video S1). Furthermore the experiments illustrate the spatial heterogeneous growth behavior of Caco-2 cells, in particular after treatment with EGF. These findings are in line with observations from corresponding white light images. However, a reliable identification of single cells and cell tracking as described previously, e. g. for epithelial cells [35], was not feasible due to the specific morphological and growth properties of Caco-2 cells.

### Detection of cellular volume, dry mass and refractive index alterations upon cytokine stimulation in single cell suspension by DHM

Zytotoxic agents such as mitomycin c can significantly impair epithelial migration and proliferation without affecting protein biosynthesis [36]. Thus, simultaneous measurements of cellular dry mass and cellular volume are of interest for characterizing the biological properties of potential drug candidates. However, as previously shown in Figure 2B, in a confluent layer, DHM analysis of individual cells was not easily possible and determination of cellular dry mass, volume and refractive index separately for single cells could not be achieved. Thus, to measure these individual parameters, we used a single cell suspension of detached cells. Quantitative DHM phase contrast images of suspended EGF- and mitomycin c-stimulated single cells as well as unstimulated control cells (*n* = 89 cells for each experiment) were analyzed as described in the section ‘*Determination of the cellular refractive index and the cell volume*’.

**Figure 3:**
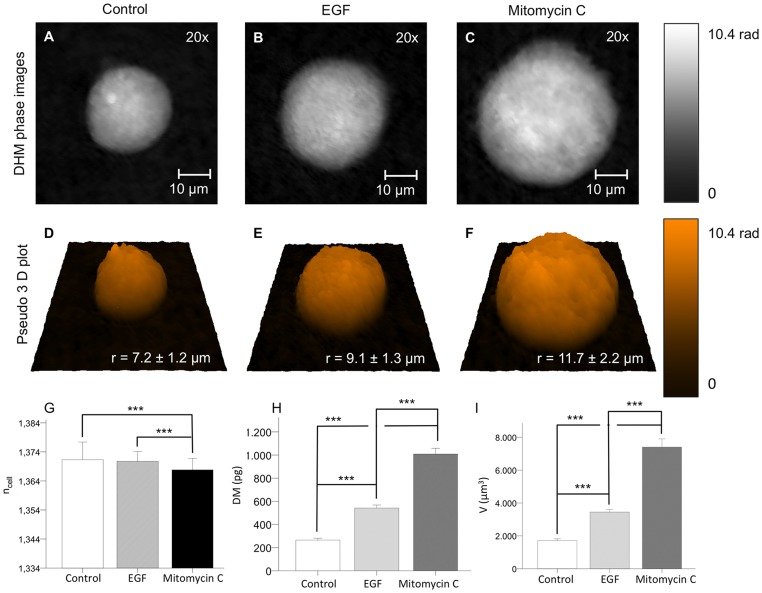
Refractive index, dry mass and cellular volume of stimulated and unstimulated Caco-2 single cells in suspension. (**A–C**) Representative quantitative DHM phase images of suspended single Caco-2 cells (coded to 256 gray levels), (**A**) untreated control cells, (**B**) after treatment with either epidermal growth factor (EGF) or (**C**) mitomycin c. (**D–F**) The me an cellular radius *r* as assessed by false color coded pseudo 3D representations of quantitative phase images was slightly increased after EGF stimulation and markedly more enhanced after mitomycin c treatment. (**G**) The refractive index *n*_cell_ of mitomycin c-stimulated cells was significantly decreased as compared to EGF-stimulated cells and untreated control cells. (**H, I**) Dry mass *DM* and cellular volume *V* of EGF-treated Caco-2 cells were significantly increased as compared to untreated cells but were reduced as compared to mitomycin c-stimulated cells. Data are means ±SE; *N* = 89, ***P<0.001 (the numerical data of diagrams G, H, I are summarized in Table S1).

Figures 3A-3C depict quantitative DHM phase images of suspended single cells (coded to 256 gray levels) with representative size and refractive index. The mean cellular radius and the cellular volume are illustrated by false color-coded pseudo 3D representations of the quantitative phase images (Figures 3D–3F). For unstimulated Caco-2 cells, a mean radius of *r_control_* = 7.2±1.2 µm was obtained (Figures 3A and 3D). In contrast, the mean radius of EGF-stimulated Caco-2 cells was found markedly enhanced (*r_EGF_* = 9.1±1.3 µm; Figures 3B and 3E) while the mean radius of mitomycin C-treated cells was even more increased (*r_mitomycin_* = 11.7±1.3 µm; Figures 3E,F). Confirming this, compared to untreated control cells, the cellular volume of EGF-stimulated cells was significantly increased (*V_control_* = 1712±108 µm^3^ vs. *V_EGF_* = 3445±168 µm^3^; *P*<0.001, Figure 3I) while the volume of mitomycin C-treated cells was quadrupled (*V_mitomycin_* = 7401±502 µm^3^ vs. *V_control_* = 1712±108 µm^3^; *P*<0.001; Figure 3I).

After stimulation with EGF, the refractive index of the treated cells was comparable to the unstimulated (1.3707±0.0004 vs. 1.3713±0.0006; Figure 3 G), in contrast to the difference seen with cellular volume. However, a significant reduction of the refractive index could be observed between mitomycin c treated cells and control (1.3678±0.0004 vs. 1.3713±0.0006; *P*<0.001, as well as between stimulated and inhibited cells (1.3707±0.0004 vs. 1.3678±0.0004; *P*<0.001).

Finally, the cellular dry mass was determined as described in section “*Analysis of cellular growth with quantitative phase microscopy*”. In accordance with the previous findings on cellular volume and refractive index, a highly significant dry mass increase of EGF- and mitomycin c-stimulated Caco-2 cells was observed in comparison to unstimulated control cells (*DM_EGF_* = 542±27 pg vs. *DM_control_* = 265±16 pg and *DM_mitomycin_* = 1009±51 pg vs. *DM_control_* = 265±16 pg; both *P*<0.001, Figure 3H). Furthermore, the dry mass increase of mitomycin c-stimulated cells was significantly higher as compared to EGF-stimulated cells *DM_mitomycin_* (1009±51) pg vs. *DM_EGF_* = 542±27 pg; *P*<0.001; Figure 3H).

Taken together, DHM was not only able to quantify morphological characteristics like volume and cell density and dry mass of Caco-2 cells but could additionally reveal alterations of these features upon stimulating and inhibiting cytokine treatment as compared to untreated control cells.

### Automated quantification of temporal development of wound closure by simultaneous monitoring of cell covered area, cell layer dry mass cell layer mean thickness and volume as well as cell number

**Figure 4:**
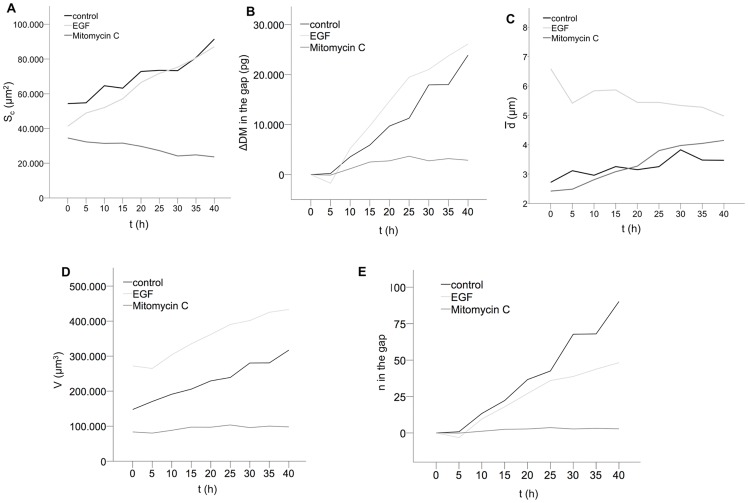
Simultaneous monitoring of cellular key characteristics during epithelial wound closure illustrated by results from a single measurement. (**A**) The cell covered area *S*_c_ after start of the wound healing assay is markedly decreased after stimulation with mitomycin c as compared to untreated control cells, and EGF-treated cells. (**B**) Over the period of 40 h, the slope of the cellular dry mass Δ*DM* of EGF-stimulated cell in the wound gap is slightly decreased in comparison to unstimulated control cells whereas mitomycin c-treated cell only reveal a modest increase in cellular dry mass. (**C**) The average cell layer thickness 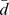 as well as temporal thickness increase of mitomycin c-treated and unstimulated control was comparable while EGF-stimulated cell show a dramatically increased cell layer thickness that slightly decreased during the observation period. (**D**) The cellular volume *V* of mitomycin c-stimulated cells was constant during the 40 h observation period. In contrast, *V* of unstimulated control cells and EGF stimulated cells increased continuously and were almost doubled after 40 h. (**E**) The quotient of total dry mass in the gap and mean dry mass of single cells for each condition reveals the absolute cell number *n* in the wound. Unstimulated and EGF stimulated cells indicated a marked increase while mitomycin c treatment resulted in an almost constant cell number.

To assess the impact and the duration of stimulating agents on epithelial wound healing, continuously monitoring of the wound closure area would be desirable. Simultaneous determination of changes in cellular dry mass and volume may give additional insights into cell cycle related processes such as cell division and cell viability [29], [30]. To this aim, in series, quantitative DHM phase images were obtained every 30 min with the setup illustrated in Figure 1 and evaluated as described in section “*Analysis of cellular growth and thickness with quantitative phase microscopy*”. Due to the spatial heterogeneous growth behavior of the Caco-2 cells (see Figure 2B) the achieved measurement data on untreated Caco-2 cells and EGF- and mitomycin c stimulated cells are illustrated in Figure 4 for results from single measurements. In Fig. 4A the relative temporal increase of the cell-covered area in comparison with the initial wound area is plotted. The area of wound closure in the control assay with untreated Caco-2 cells could be successfully determined over a 40 h period to Δ*S_c, control_* = 37139 µm^2^. In the assay with EGF- and mitomycin c-treated cells, increased would closure of EGF-stimulated cells as compared to mitomycin- inhibited cells could be observed (area increase of Δ*S_c, EGF_* = 45782 µm^2^ vs. area decrease Δ*S_c, mitomycin_* = -10926 µm^2^. Surprisingly, the area increase of EGF stimulated cells was comparable to the increase of control cells. Figure 4B shows the relative development of the cellular dry mass Δ*DM* obtained from the same DHM phase images. This was performed by subtracting the initial dry mass value that was calculated from the first quantitative phase image from all subsequently measured dry mass values of each time-lapse series. For untreated Caco-2 cells Δ*DM_control_* = 23.9 ng was obtained at 40 h after start of the experiment (Figure 4B). Altered dry mass amounts were also detected with DHM within 40 h after start of EGF- and mitomycin c-treatment (Δ*DM_EGF_* = 26.1 ng and Δ*DM_mitomycin_* = 2.9 ng; Figure 4B). Furthermore, a higher dry mass increase for EGF stimulated cells than for control cells were observed. Figure 4C depicts the corresponding temporal relations of the mean cellular thickness 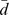 of the assays that were calculated by using Eq. 3 using the mean refractive indices obtained from suspended cells (see section “*Determination of cellular volume, dry mass and refractive index alterations upon cytokine stimulation in single cell suspension*”) and *n_medium_* = 1.339. For control cells, the increase of 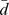 was nearly linear. Similarly, a linear increase of mytomycin c-treated cells was observed while the mean thickness of EGF-stimulated cells was slightly decreased during the observation period. In contrast to the results in Figures 4A–4C, no difference of 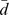 was observed between mitomycin c-inhibited cells and control cells while the mean thickness of EGF-stimulated cell layers was almost doubled. In order to calculate the mean volume *V* of the cell layers, the absolute data for the area *S_c_* covered by the cells in Figure 4A and the mean cell thickness 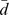 in Figure 4C were multiplied (see Figure 4D). The cell layer volume of unstimulated Caco-2 cells increased within the observational period and was determined to be 317147 µm^3^ at the end of experiment. In correspondence to Figure 4A, the cell layer volume increase of EGF-treated cells was comparable to the control cells, while mitomycin c-stimulated cells showed a constant cell layer volume over time (40 h: 433280 µm^3^ vs. 97994 µm^3^). The similar increase of *S_c_* and cell volume for EGF stimulated cells and control cells may be explained by inhibitory effects of mitomycin c on the EFG stimulated cells, as stimulated and inhibited cells were observed in a single assay with the same cell culture medium.

Finally, we estimated the total number of cells in the wound gap by dividing the relative dry mass (Figure 4B) through the mean single cell dry mass retrieved from the suspended single cells (Figure 3H). In Figure 4E the resulting temporal relation of the total cell number in the wound is plotted. The results in Figure 4E show that DHM is able to provide a reliable automated assessment of the number of cells in the wound. In addition, results for EGF stimulated cells indicate marked enhancement of cells in the wound gap as compared to the number of mitomycin c-inhibited cells which was constant. In line with the previous findings, after 40 h a slightly higher number of control cells than for EGF stimulated cells was found in the wound.

**Figure 5:**
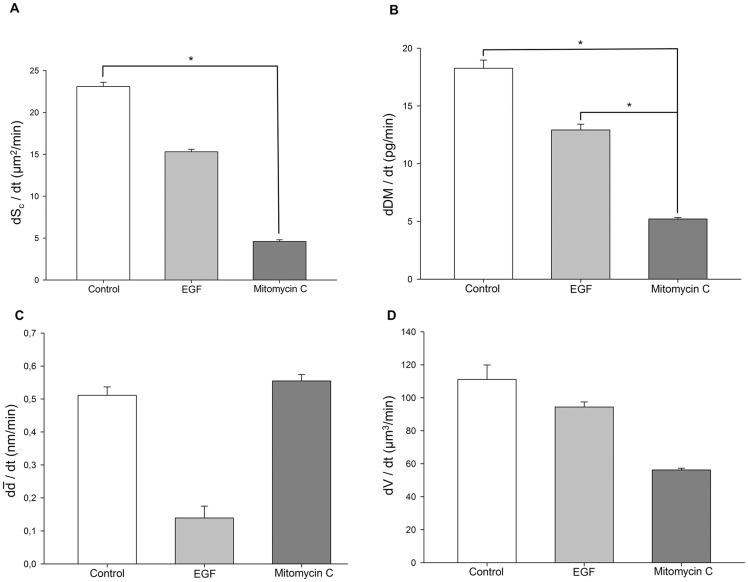
Time constants of key cellular characteristics during epithelial wound closure. (**A**) While the change of cell-covered area per minute 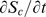 of mitomycin c-stimulated cells was significantly diminished as compared to untreated control cells, no significant difference was detected between EGF- and untreated control cells. (**B**) The temporal dry mass change 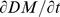 of mitomycin c-stimulated cells was significantly decreased as compared to EGF-stimulated and untreated control cells. (**C,D**) No significant differences in the temporal changes of cell layer thickness 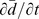 and cellular volume 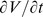 were detected between EGF or mitomycin c-stimulated cells and untreated control cells. Data are means ±SE; *, *P*<0.05; (the numerical data of the diagrams A–D are summarized in Table S2).

All temporal dependencies in Figure 4 show a mainly linear temporal dependency. Therefore, the mean change of the cell covered area, the dry mass, the mean thickness of the cell layer and the cell volume per minute were determined by linear regression from averaged data of three independent experiments (Figure 5). For the assay with unstimulated control cells, the area increase was obtained to be 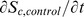 = 23.1±0.5 µm^2^/min. A significantly enhanced area increase after EGF stimulation as compared to mitomycin c treatment was observed (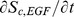 = 15.3±0.3 µm^2^/min vs. 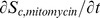 = 4.6±0.2 µm^2^/min; *P* = 0.01; Figure 5A). Fig. 5B shows the dry mass change per minute for untreated Caco-2 cells which was determined to 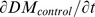 = 18.3±0.7 pg/min. With regard to cytokine-stimulated cells, a significantly increased dry mass change of EGF-treated cells when compared to mitomycin c-stimulated cells could be corroborated (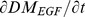 =  (12.9±0.5) pg/min vs. 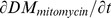 = 5.2±0.1 pg/min; *P*<0.01; Figure 5B). Finally, for untreated control cells, the cell layer thickness was 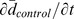 = 0.51±0.03 nm/min (Figure 5C) while a volume change of 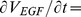
111±9 µm^3^/min was retrieved (Figure 5D). No statistical difference was observed regarding the change of cell layer thickness and volume per minute between EGF- and mitomycin c-stimulated cells (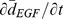 =  (0.14±0.04 nm/min vs. 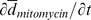 = 0.55±0.02 µm/min; and 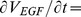
94±3 µm^3^/min vs. 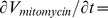
56±1 µm^3^/min, Figure 5C,D). Finally, it has to be mentioned that the average values in Figure 5 for the area change rate and the thickness change rate appear partly to be in disagreement with the results from the single measurement in Figure 4. This is caused by the heterogeneous growth behavior of the Caco-2 cells in the underlying individual DHM measurements (see illustration in Fig. 2B) and also demonstrates the need for a sufficient number of independent measurement repetitions to achieve statistically reliable data sets under the conditions of our experiments.

### Alteration of cellular thickness of stimulated and inhibited Caco-2 cells

**Figure 6:**
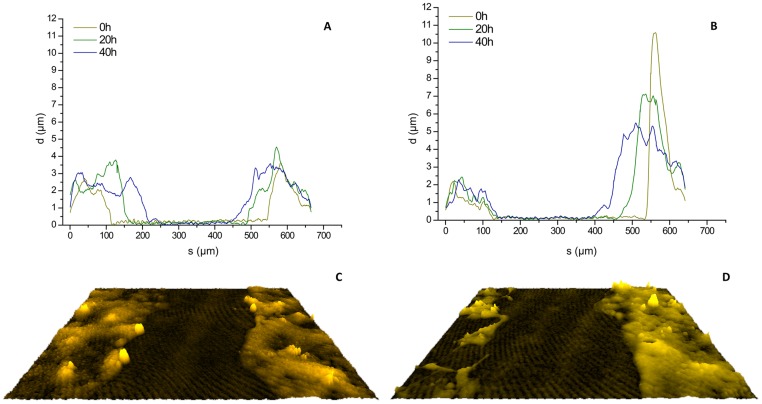
Alteration of cellular thickness of stimulated Caco-2 cells during wound healing. (**A**) Averaged profiles *S* through the cell layer thickness *d* of control cells and (**B**) cell layers after treatment with mitomycin c (left) and EGF (right). (**C,D**) False color-coded pseudo 3D plots of corresponding representative quantitative DHM phase contrast images.

Finally, DHM examinations also allowed assessing spatial changes in cellular thickness during the wound healing process. Figure 6A shows averaged profiles through the cell thickness of untreated control cells at 0, 20 and 40 h. Additionally, Figure 6B depicts averaged profiles from mitomycin c-inhibited cells (*left*) and EGF-stimulated cells (*right*) from a single representative single experiment. Upon stimulation with EGF, cellular thickness of Caco-2 cells was markedly increased as compared to mitomycin c-treated cells. Figures 6C,D and Video S2 illustrate these finding by false color coded pseudo 3D images of representative quantitative DHM phase contrast images.

## Discussion

In this study, we prove DHM to enable continuous, stain-free monitoring of intestinal epithelial wound healing *in vitro* and to provide simultaneous quantification of key cellular characteristics such as cell volume, cell thickness, dry mass and cell density which may help to characterize therapeutic effects of potential drug candidates.

Proliferation and migration are two major steps required for successful wound closure following ulceration and inflammation [1] and numerous agents have been proposed to stimulate wound healing [37], [38]. Preclinical evaluation of potential drug candidates *in vitro* is traditionally performed by the use of mechanically induced wounds and healing assessed by the number of cells beyond the wound edge [39]–[42]. However, this experimental approach is limited due to its inability to discriminate migrating from proliferating cells and the necessity of staining to identify cell borders, which excludes repetitive measurements of migrating cells [10], [11]. While acceleration of epithelial migration benefits wound closure [43], enhanced proliferation may be associated with adverse side effects such as malignant transformation and morphological changes. In our hands, quantitative DHM phase contrast images in combination with time lapse analysis allowed quantification of the cell-covered area as well as accurate identification of proliferation cells by quantification of cellular dry mass and morphology (Figure 2, Video S1). During proliferation of Caco-2 cells, a significant increase of protein amount has been described previously [44]. Notably, the protein amount within a single cell generates up to 80% of the total dry mass of the cell [45] and whole protein amount of a cell population correlates well with the number of cells [46]. By using suspended cells, we were able to determine dry mass and volume for single cells. Interestingly, we observed a moderate increased in dry mass upon EGF stimulation as compared to the dry mass of unstimulated cells while mitomycin c treatment resulted in highly significantly increased cellular dry mass. This finding is in line with a study by Mir *et al.* demonstrating that human osteosarcoma U2OS cells double their dry mass before entering mitosis and detecting that daughter cells possess exactly one half of the parental cell mass [47]. The high increase in dry mass of mitomycin c-treated cells is most likely the result of the mitosis inhibitory effect of this agent [48]. Consequently, the wound closure of EGF-treated cells was accelerated as compared to untreated cells and cells treated with mitomycin c.

In [47], it is reported that the cellular dry mass is directly dependent on the growth rate. Moreover the accuracy of optically assessed cellular dry mass by quantitative phase imaging as well as the possibility to quantify cell growth noninvasively by optical imaging alone has nicely been demonstrated earlier [49]. Furthermore, non-invasive DHM measurement of dry mass can be performed repetitively from living cells and may be continued over a longer period of time as we demonstrate [21]. In addition, the DHM-based approach for determination of the dry mass is independent of the intracellular water content which may be influenced by the osmolality of the cell culture medium or the tested agent [50]. Importantly, simultaneous determination of cellular dry mass changes during wound healing *in vitro* is not provided by traditional approaches.

There is evidence, that wound healing is significantly influenced by the environment, surrounding cells and tissues. Recently, it was demonstrated that deformation and extracellular pressure can stimulate intracellular enzyme activity [51], [52] and may influence cell proliferation and healing. For example, in a murine model, intestinal obstruction resulted in decreased wound healing of chemically induced mucosal ulcers [53]. Thus, beside measurement of migration and proliferation, a comprehensive evaluation of epithelial wound healing requires the assessment of cellular morphological features such as thickness. Interestingly, cellular thickness may also be used to evaluate proliferation since doubling of dry mass and increase in cell size is routinely observed before each division [54].

Recently, Pavillon *et al.* demonstrated that DHM may also be used to differentiate between apoptosis and necrosis by assessment of cellular volume [22]. While apoptosis is initially associated with a reduced cellular volume, necrosis is characterized by a significant increase of cellular volume prior the cell collapse. It has been shown that alterations of cellular volume are modulated by ionic pathways [55], [56] and changes of cellular volume as assessed by DHM were successfully linked to apoptotic rates in murine cortical neurons *in vitro* [22]. Moreover, toxic effects of methanol were detected by optical thickness measurements since altered optical thickness of epithelial HeLa cells was indicative of pyknosis within these cells [24]. Similarly, Kühn *et al*. demonstrated DHM to be ready to use for assessment of cytotoxicity and cell viability by determination of morphological and biomolecule (protein and nucleic acid) changes. The authors additionally report DHM to be a magnitude faster than automated standard fluorescence microscopy [57].

It should be mentioned that our results revealed that EGF-stimulated Caco-2 cells grow with a similar dry mass rate like untreated control cells but show different morphological features which is indicated by a significant difference in the cell layer thickness. These findings may be explained by the specific design of our experiments in which EGF and mitomycin c were applied in a single assay. Nevertheless, the obtained results also demonstrate that the accuracy of the applied DHM method is sufficient to detect, for example interference of different cytokine in an assay.

In summary, our results demonstrate that DHM provides several advantages with regard to previously established methods for monitoring of wound healing *in vitro*. Although the detection of individual cells is limited as successful identification depends strongly on the morphological properties of the individual cell type, DHM can continuously quantify minimally invasive proliferation and migration of an ensemble with only low exposure of the sample to light. Furthermore, DHM is able to distinguish between both processes by biophysical information that is retrieved by optical path length changes without molecular markers. In addition, DHM can detect cellular hypertrophy as well as atrophy and due to the working principle of DHM, direct monitoring of cell motility and simultaneous determination of their key morphological characteristics can be performed. Moreover, the use of DHM may not be limited to monitoring of mammalian cells but was has already been shown feasible for assessment of bacteria [58], yeast cells [59] or parasites [27]. Finally, traditional methods often measure one or a few parameters simultaneously, which may impair the statistical power of the experiments. In contrast, DHM allows retrieval of several parameters in parallel.

### Conclusions

In conclusion, we propose that the above-presented parameters give significantly advanced insights into cellular characteristics during wound healing in *in vitro* assays using label-free quantifying proliferation and migration with biophysical parameters. As DHM is able to simultaneously assess cellular characteristics by continuously monitoring and quantifying cell migration, morphological alterations and proliferation, DHM can assist in the evaluation of potential therapeutics, help elucidate the specific role of certain cytokines for wound healing, and help dissect cellular alterations which may be related to distinct cellular functions, enabling investigators to perform automated, cost-efficient and minimally invasive quantitative assays with minimized sample interaction in a flexible and more sophisticated way.

## Materials and Methods

### Cell culture

Caco-2 cells (passages 22-28) (purchased from ATCC, Manassas, VA, USA) were cultured in Dulbecco's Modified Eagle Medium (DMEM, Gibco-Invitrogen, Cergy Pontoise, France) supplemented with 20% foetal bovine serum (FBS; Gibco-Invitrogen), 1% non-essential amino acids and 1% penicillin/streptomycin in a 5% CO_2_, 95% humidity environment at 37°C. Cells were seeded on ibidi dishes (35 mm with high culture-insert coating) at a density of 4×10^5^ cells/cm^2^ for wound healing assay. Three days after seeding, the medium was changed every third day. Experiments were conducted on the 4th day of culture.

### Cell layer wound assays

*In vitro* wound assays were performed using IBIDI Culture-Inserts according to Shih *et al*. [60]. Briefly, when confluent monolayers of Caco-2 cells were established on ibidi dishes (35 mm with high culture-insert coating), cells were washed twice with phosphate buffered saline (PBS) to remove residual cell debris. In a first set of experiments, untreated Caco-2 cells were investigated to prove feasibility of DHM to accurately monitor wound healing and determine morphological cell characteristics. Furthermore, to evaluate the ability of DHM to detect alterations in wound healing behaviour upon stimulating or inhibiting cytokine exposure, cells were treated either with epidermal growth factor (EGF) [48] or mitomycin c [34] in a single assay. In detail, wounded monolayers were then cultured for 24 h in fresh serum-deprived medium (0.1% FBS) supplemented with 20 ng EGF/ml serum [61] or 2 µg mitomycin c/ml serum. Cells treated with medium alone served as controls. Subsequently, culture inserts were removed and holograms of the remaining gaps were taken for 40 h. Migration and proliferation were measured as described below. For examination of *in vitro* wound healing assays with DHM, the cell culture medium was replaced by HEPES (4-(2-hydroxyethyl)-1-piperazineethanesulfonic acid) buffered medium (20 mM HEPES) and Caco cells were observed in time-lapse series for 40 h with the experimental setup described in section ‘*Quantitative phase imaging with digital holographic microscopy*’. Digital holograms were recorded every 30 min. The obtained quantitative DHM phase contrast images were further evaluated to quantify cell proliferation and migration as described in section ‘*Cell proliferation rates during wound healing*’. Experiments were repeated in triplicate as a minimum.

### Quantitative phase imaging with digital holographic microscopy

An inverted microscope (iMIC, Till Photonics, Gräfelfing, Germany) with an attached DHM module based on a principle described in [16] was applied for bright field imaging and quantitative DHM phase contrast imaging. Figure 1 shows the scheme of the experimental setup and illustrates the utilized off-axis DHM configuration. The coherent light source was a frequency-doubled Nd: YAG laser (*λ* = 532 nm, Coherent Compass 315M, Coherent, Luebeck, Germany). The cell cultures were observed in special Petri dishes for wound healing observations (ibidi µ-Dish with culture-Insert, ibidi GmbH, Munich, Germany). The sample was illuminated with laser light in transmission (object wave) and imaged by a microscope lens and a tube lens on a charge-coupled device camera (DMK 41BF02, The Imaging Source, Bremen, Germany). The object wave was superimposed with the slightly tilted reference wave for the generation of off-axis holograms which were recorded by the camera sensor and transferred to an image-processing system using custom built C++ based hologram acquisition software. For imaging of adherent cells during wound healing a 10× microscope lens (Zeiss EC Plan-Neofluar 10×0.3, NA = 0.3) was utilized while suspended cells were observed with a 20× microscope lens (Zeiss LD Achroplan 20x/0.4 Korr, NA = 0.4).

The reconstruction of the digitally captured holograms was performed by spatial phase shifting reconstruction in combination with optional holographic autofocusing as described with details elsewhere (see [16], [62], [63] and included references). The resulting quantitative phase images quantify the change of the optical path length delay caused by thin mainly transparent (phase) objects such as the investigated living cell cultures. The cell induced phase contrast *Δφ* depends on the cell thickness d, the integral cellular refractive index *n_cell_*, the refractive index *n_medium_* of the cell culture medium [16], [18] and the wavelength *λ* of the laser light used in the DHM system:
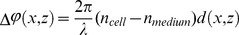
(1)


### Determination of the cellular refractive index and the cell volume

The determination of the cell thickness with Eq. 1 requires information about the integral cellular refractive index *n_cell_*. In addition, *n_cell_* quantifies the cell density as it is directly related to the intracellular solute concentration [64]. Also the cellular volume (*V*) can be related to the cellular response to drugs. Thus, *n_cell_* and *V* were analyzed for inhibited and stimulated Caco-2 cells as well as for untreated control cells. For determination of *n_cell_* and *V*, cells were detached (trypsinized) and suspended in cell culture medium with an osmolality of 320 mOsmol/kg in petri dishes (ibidi µ-dishes GmbH, Martinsried, Germany). For each sample, digital holograms of *N* = 89 selected single cells with spherical appearance were recorded. From the resulting quantitative DHM phase contrast images, the integral cellular refractive index *n_cell_* and *V* were determined by numerical fitting of Eq. (1) as described with details previously [65].

### Analysis of cellular growth and thickness with quantitative phase microscopy

In order to analyze cellular growth and morphology changes, three parameters were calculated from quantitative DHM phase contrast images. First the area *S_c_* covered by the cells was determined in quantitative DHM phase images by image segmentation using the free software cell profiler (www.cellprofiler.org, [66]. Then the averaged phase contrast 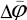 caused by the cells in the area *S_c_* was calculated.

In addition, from 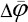 and 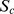 the cellular dry mass *DM* was retrieved [29], [30]:
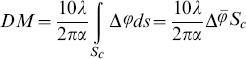
(2)following approaches as described in [47], [59]. For the parameter *α*, the value 0.002 m^3^/Kg was estimated.

Furthermore, from 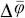 and *n_cell_* and *n_medium_* and Eq. (1) the average thickness 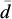 was determined from Eq. (1):
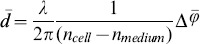
(3)


### Statistical analysis

Data were analyzed using SPSS 20.0 (IBM). Results are expressed as means ± standard error. Using the Mann-Whitney U-test and the χ-squared test appropriate comparisons between different data sets were performed. *P*-value <0.05 was considered to be statistically significant.

## Supporting Information

Table S1 **Numerical data of suspended CaCo-2 single cells displayed in** **Figure 3** **.**
(DOCX)

Table S2 **Numerical data of temporal relations displayed in** **Figure 5** **.**
(DOCX)

Video S1 **Movie of segmented quantitative DHM phase images during epithelial wound healing** ***in vitro*** **.** Left: CaCo-2 cells inhibited with mitomycin c, right: CaCo-2 cells stimulated with epidermal growth factor (EGF).
(MP4)

Video S2 **False color-coded pseudo 3D images of representative quantitative DHM phase contrast images during epithelial wound healing** ***in vitro*** **.** Left: CaCo-2 cells inhibited with mitomycin c, right: CaCo-2 cells stimulated with epidermal growth factor (EGF).
(WMV)
