# Supplementary material for: Quantitative Stain-Free and Continuous Multimodal Monitoring of Wound Healing In Vitro with Digital Holographic Microscopy
Source: PLoS One. 2014 Sep 24;9(9):e107317. doi: 10.1371/journal.pone.0107317 (PMC4174518; doi:10.1371/journal.pone.0107317)
Supplement: Table S1 — Numerical data of suspended CaCo-2 single cells displayed in Figure 3 . (DOCX) [file pone.0107317.s001.docx]

**Supporting Table S1:** Numerical data of suspended CaCo-2 single cells displayed in Figure 3**.**

*n*_cell_ : average cellular refractive index (Fig. 3G), *DM*: average dry mass (Fig. 3H) and *V*: average cell volume (Fig. 3I) of suspended Caco-2 single cells (mean ± standard error)

| **sample treatment** | ***N*** | ***n*_cell_** | ***DM* (pg)** | ***V* (µm^2^)** |
| --- | --- | --- | --- | --- |
| untreated (control) | 89 | 1.3713 ± 0.0006 | 265 ± 16 | 1712 ± 108 |
| EGF | 89 | 1.3707 ± 0.0004 | 542 ± 27 | 3445 ± 168 |
| mitomycin c | 89 | 1.3678 ± 0.0004 | 1009 ± 51 | 7401 ± 502 |

EGF: epidermal growth factor, *N*: cell number
