# Supplementary material for: Quantitative Stain-Free and Continuous Multimodal Monitoring of Wound Healing In Vitro with Digital Holographic Microscopy
Source: PLoS One. 2014 Sep 24;9(9):e107317. doi: 10.1371/journal.pone.0107317 (PMC4174518; doi:10.1371/journal.pone.0107317)
Supplement: Table S2 — Numerical data of temporal relations displayed in Figure 5 . (DOCX) [file pone.0107317.s002.docx]

**Supporting Table S2:** Numerical data of temporal relations displayed in Figure 5.

$\partial S_{c}/\partial t$: average rate of area change (Fig. 5A), $\partial DM/\partial t$(ng/min): average rate of dry mass change (Fig. 5b), $\partial\bar{d}/\partial t$: average rate of thickness change (Fig. 5c), $\partial V/\partial t$: average rate of volume change (Fig. 5D). Each value was obtained by linear regression from 222 data point that were retrieved in three independedly performed experiments.

| **sample treatment** | $\partial S_{c}/\partial t$ **(µm^2^/min)** | $\partial DM/\partial t$**(ng/min)** | $\partial\bar{d}/\partial t$ **(nm/min)** | $\partial V/\partial t$ **(µm^3^/min)** |
| --- | --- | --- | --- | --- |
| untreated (control) | 23.1 ± 0.5 | 18.3 ± 0.7 | 0.51 ± 0.03 | 111 ± 9 |
| EGF | 15.3 ± 0.3 | 12.9 ± 0.5 | 0.14 ± 0.04 | 94 ± 3 |
| mitomycin c | 4.6 ± 0.2 | 5.2 ± 0.1 | 0.56 ± 0.02 | 56 ± |

EGF: epidermal growth factor
